# Frontiers in Dissecting and Managing *Brassica* Diseases: From Reference-Based RGA Candidate Identification to Building Pan-RGAomes

**DOI:** 10.3390/ijms21238964

**Published:** 2020-11-25

**Authors:** Yueqi Zhang, William Thomas, Philipp E. Bayer, David Edwards, Jacqueline Batley

**Affiliations:** School of Biological Sciences, The University of Western Australia, Perth 6009, Australia; 22078946@student.uwa.edu.au (Y.Z.); william.thomas@research.uwa.edu.au (W.T.); philipp.bayer@uwa.edu.au (P.E.B.); dave.edwards@uwa.edu.au (D.E.)

**Keywords:** *Brassica*, RGA, reference-based QTL mapping, GWAS, RGASeq, pan-RGAome

## Abstract

The *Brassica* genus contains abundant economically important vegetable and oilseed crops, which are under threat of diseases caused by fungal, bacterial and viral pathogens. Resistance gene analogues (RGAs) are associated with quantitative and qualitative disease resistance and the identification of candidate RGAs associated with disease resistance is crucial for understanding the mechanism and management of diseases through breeding. The availability of *Brassica* genome assemblies has greatly facilitated reference-based quantitative trait loci (QTL) mapping for disease resistance. In addition, pangenomes, which characterise both core and variable genes, have been constructed for *B. rapa*, *B. oleracea* and *B. napus*. Genome-wide characterisation of RGAs using conserved domains and motifs in reference genomes and pangenomes reveals their clustered arrangements and presence of structural variations. Here, we comprehensively review RGA identification in important *Brassica* genome and pangenome assemblies. Comparison of the RGAs in QTL between resistant and susceptible individuals allows for efficient identification of candidate disease resistance genes. However, the reference-based QTL mapping and RGA candidate identification approach is restricted by the under-represented RGA diversity characterised in the limited number of *Brassica* assemblies. The species-wide repertoire of RGAs make up the pan-resistance gene analogue genome (pan-RGAome). Building a pan-RGAome, through either whole genome resequencing or resistance gene enrichment sequencing, would effectively capture RGA diversity, greatly expanding breeding resources that can be utilised for crop improvement.

## 1. Agricultural Importance of *Brassica* Species

The *Brassica* genus belongs to the Brassicaceae family and *Brassica* crops are cultivated as oilseeds, vegetables, condiments and forages [1]. Commonly cultivated *Brassica* species include the diploids *B. rapa* (AA genome: 2n  =  2×  =  20), *B. nigra* (BB: 2n  =  2×  =  16) and *B. oleracea* (CC: 2n  =  2×  =  18), and the allotetraploid species *B. juncea* (AABB: 2n  =  4×  =  36), *B. napus* (AACC: 2n  =  4×  =  38) and *B. carinata* (BBCC: 2n  =  4×  =  34). The triangle of U summarises the genomic relationship of these three important amphidiploid hybrids and their diploid progenitor species [2]. *B. rapa* originated in the Mediterranean and central Asia and is commonly known as turnip and Chinese cabbage, but has subspecies also cultivated for fodder and rapeseed [3]. Black mustard (*B. nigra*) seed is commonly used as a spice and condiment [4], whilst *B. oleracea* is mainly used as a vegetable crop, including brussels sprouts, cabbage, cauliflower, broccoli and Chinese kale [5]. *B. napus*, or canola, is the third most important oilseed crop globally following soybean and palm [6]. *B. juncea* is cultivated as an oilseed, spice, vegetable and fodder crop species [7] and *B. carinata* originated from the Ethiopian plateau and is an important condiment, vegetable and oilseed crop in northeast Africa [8]. 

Several diseases hinder the production of *Brassica* crops, for example, blackleg disease, caused by the dothideomycete fungus *Leptosphaeria maculans,* is a major constraint to canola production in Australia, Europe and America [6]. The disease causes 10–20% average annual yield losses in Australia, Canada and the UK [9,10,11]. In addition, serious epidemics have destroyed entire crops in regions of France in 2000 [12] and in the Lower Eyre Peninsula, Australia, in 2003 [13]. Stem rot caused by *Sclerotinia sclerotiorum* leads to yield losses in canola production worldwide [14]. An average sclerotinia stem rot incidence of 10–20% has been reported in Canadian provinces each year, with an incidence of 94% within an individual canola crop recorded in Saskatchewan in 2010 [14]. The disease can also cause up to 80% yield loss in China [15]. Clubroot, caused by *Plasmodiophora brassicae*, offers challenges to crop production worldwide [16]. The disease led to 50% yield loss in cruciferous crops in Canada [17] and 20–30% yield loss in China [18]. In addition, turnip mosaic virus, downy mildew caused by the oomycete pathogen *Hyaloperonospora brassicae*, fusarium wilt caused by the fungus *Fusarium oxysporum* f. sp. *conglutinans* and black rot caused by the bacteria *Xanthomonas campestris* pv. *campestris* are important pathogens which affect *Brassica* crops worldwide [1]. 

## 2. Quantitative and Qualitative Disease Resistance Mechanisms in Plants

Quantitative disease resistance is underpinned by multiple genes of small effect which provide incomplete or partial resistance in plants [19]. The pathogen-associated molecular pattern (PAMP)-triggered immunity (PTI) contributes to quantitative resistance [20]; transmembrane pattern recognition receptors (PRRs), including receptor-like kinase (RLKs) and receptor-like proteins (RLPs), are involved in PTI and recognise pathogen- or microbe-associated molecular patterns (PAMPs/MAMPs). PTI is also important in nonhost resistance whereby most plants are resistant to most microbial pathogens. 

PRRs function collaboratively in mediating defence signalling [21]. Many PRRs rely on the regulatory protein brassinosteroid insensitive 1-associated receptor kinase 1 (BAK1) and others on the somatic embryogenesis receptor-like kinases (SERKs) [22]. Some RLKs, such as the RLK suppressor of BIR1 (SOBIR1), associate with RLPs to transmit the signal to downstream components [23]. Several receptor-like cytoplasmic kinases (RLCKs) link the PRR complex to downstream defences, triggering a number of cellular events including an increase of cytoplasmic calcium cation and anion effluxes, and extracellular alkalisation, the production of reactive oxygen species (ROS), and the activation of MAP kinase (MPK) cascades [24]. Some RLKs play a role in both defence and developmental pathways such as BAK1 [21]. Many pathogen effectors impede PRR functions by targeting microbial patterns, PRRs or components in the signalling cascade or interfere with RLKs that regulate plant growth and development to increase plant susceptibility. 

Qualitative disease resistance is underpinned by major genes which provide complete or near-complete resistance in plants [19]. The effector-triggered immunity (ETI) response commonly forms the basis of qualitative resistance in plants [20]. ETI is responsible for host-mediated defence against adapted pathogens by recognising specific effector (avirulence) proteins secreted into host cytoplasm by the pathogen. Qualitative resistance genes typically encode nucleotide-binding-site leucine-rich repeat (NLR) proteins; however, RLK types have also been identified [19]. The recognition of effectors by NLRs also leads to the accumulation of ROS, activation of MAP kinase cascades, and defence gene expression [25]. In addition, NLR activation manifests as a hypersensitive response whereby localised cell death occurs at the site of penetration by biotrophic pathogens. 

The most abundant resistance gene family encodes the intracellular receptor proteins NLR, which are functionally analogous to NOD-like receptors in the animal innate immune system [20]. Canonical NLR genes are comprised of a conserved central NB-ARC (nucleotide-binding adaptor shared by APAF-1, certain R gene products and CED-4) domain, a leucine-rich repeat (LRR) domain, varying in the number of repeats, at the C-terminus and a TIR (homologous to the Drosophila TOLL and human interleukin-1 receptor) or CC (coiled-coil) or CCr (a zinc finger or resistance to powdery mildew 8 (RPW8)) motif at the N-terminus. Based on this N-terminus motif NLRs are categorised into three subclasses TIR-NBS-LRR (TNL) CC-NBS-LRR (CNL) and RPW8-NBS-LRR (RNL).

PRRs function not only in immune response, but also regulation of plant growth and development, reproduction and symbiosis [21]. Plant RLKs, analogous to animal receptor tyrosine kinases, contain an ectodomain (ECD), transmembrane domain and a cytoplasmic kinase domain, while RLPs lack the kinase domain [21]. The ECD domain is highly variable allowing the recognition of a wide range of ligands including steroids, peptides, polysaccharides and lipopolysaccharides. RLKs can be classified into RD (arginine I and aspartate (D) amino acid residues at the activation site of the kinase domain) and non-RD types based on the conservation of the arginine residue, among which non-RD kinases are mainly involved in plant immunity [26]. 

## 3. Genome/Pangenome-Wide RGA Prediction in *Brassicas*

The advance in next generation sequencing technologies have facilitated the construction of multiple genome assemblies for *B. rapa* [27,28], *B. oleracea* [28,29,30,31], *B. napus* [32,33,34,35] and *B. juncea* [36,37]. The concept of the pangenome was proposed as the new reference genome beyond a single reference for a species [38]. Pangenomes represent the species-wide genome diversity, which includes core genes present in all individuals and variable genes absent in some individuals. Pangenomes for *B. oleracea* [39] and *B. napus* [40] have also been constructed. 

The availability of genome and pangenome assemblies of several *Brassica* species have facilitated the genetic study of disease resistance mechanisms. RGAs can be predicted bioinformatically based on their conserved domains and motifs [41]. A number of homology-based computational methods have been applied to annotate RGAs using conserved motifs, domains and recognised structural features [20]. These methods are based on BLAST similarity searches (Pfam and InterProScan), sequence alignment with resistance gene databases (PRGdb), searches for domain sequence homology with Hidden Markov Models (HMMER) or motif discovery (MEME). An integrative RGA prediction pipeline, RGAugury, was developed to firstly identify conserved domains and motifs including NB-ARC, LRR, transmembraI (TM), serine/threonine and tyrosine kinase (STTK), lysin motif (LysM), CC and TIR, and then classify these domains into four major RGA families; NBS-encoding, TM-CC, and membrane-associated RLP and RLK [41]. Genome-wide RGA repertoires characterised in *Brassica* genome and pangenome assemblies are comprehensively summarised in Table 1. 

### 3.1. B. rapa

The first characterisation of NLRs in *B. rapa* was based on bacterial artificial chromosome (BAC) clones of *B. rapa* ssp. *pekinensis cv. Chiifu* covering around 19–27% of the genome and 47–66% of the euchromatic gene space. As an incomplete assembly was used, only 92 NLRs were identified, using a BLASTP search of the NBS domains queried with 207 *A. thaliana* NLR genes from the NIBLRRS server, and verified with sequences from 26,416 unigenes and 127,144 expressed sequence tag (EST) sequences of *B. rapa* ssp. *pekinensis* cv. Chiifu downloaded from the KBGP database (The Korea *B. rapa* Genome Project) [42]. In comparison, many more NLRs were predicted in the *Chiifu-401-42* v.1 genome assembly [43,44,45,46]. Three studies identified similar numbers of NLRs, 206 [43], 204 [44] and 202 [46], in the *B. rapa Chiifu-401-42* v.1 assembly using BLAST and/or HMMER search with the Pfam NB-ARC domain (PF00931). In contrast, more NLR genes (249) were predicted using MAST/MEME (Motif alignment and search tool/Multiple em for motif elicitation) and InterProScan analyses [45]. A total of 303 RLK genes were predicted in the *B. rapa Chiifu-401-42* v.1 genome using Pfam IDs retrieved by searching in the Pfam database with previously published *A. thaliana* LRR-RLK protein sequences [47]. 

### 3.2. B. oleracea

A total of 157 NLRs were identified in the *B. oleracea* TO1000 v1 genome assembly through searching with the Pfam NB-ARC domain (PF00931) using the HMMER V3.0 programme [43]. Using HMMER v3.1b2 based on a different cultivar; *B. oleracea capitata* homozygous line 02–12, 138 NLR genes were identified [48]. A similar number of NLRs (146) were identified in the TO1000 v1 and *capitata* homozygous line 02–12 assemblies using HMMER V3.0 [46]. Applying the MEME/MAST and InterproScan approach, many more NLR genes (443) were identified in the same genome assembly [45]. Based on the improved assembly TO1000 v2.1 and using the RGAugury pipeline version 2017-10-21, Bayer et al. [5] identified 439 NLR, 822 RLK and 159 RLP genes. RGAs had clustered arrangements. For example, 27 gene clusters harbouring 81 NLRs which represented 55.48% of the total NLRs were clustered in the *B. oleracea* TO1000 v1 and *capitata* homozygous line 02–12 assemblies [46]. 

In comparison with predictions based on single genome assemblies, more RGAs with diverse patterns are predicted in the *B. oleracea* pangenome which contains nine cultivars (broccoli, brussels sprouts, cabbage, cauliflower, kale and kohlrabi) and the wild relative *B. macrocarpa* [5]. There are variations in abundance of RGA and genetic diversities among cultivars. Among the total of 556 NLRs, 901 RLKs and 213 RLPs in the pangenome, TO1000 had the lowest number while the wild relative *B. macrocarpa* had the highest number of the three RGA classes. Of all the RGA genes, 1231 genes were core and 167 were variable. 

### 3.3. B. napus 

Among the *B. napus* assemblies which contain predicted NLRs, RLPs and RLKs, Darmor-*bzh* v4.1 has the highest number of RGAs; a total of 621 NLRs, 1497 RLKs and 273 RLPs were identified in the Darmor-*bzh* v4.1 assembly using the RGAugury pipeline [50]. Several other studies only predicted NLRs [32,45,46] or RLPs [49]. The variations of the number of RGAs predicted in the four studies compared with that of Tirnaz et al. [50] based on the same genome assembly was likely due to differences in the prediction methods. For example, Alamery et al. [45] deployed MEME/MAST and InterProScan approaches, Fu et al. [46] made the prediction using HMMER followed with verification on the Pfam website, and Stotz et al. [49] used the MEME/MAST approach for prediction and the putative RGAs were verified with Pfam, TMHMM, Phobius and SMART searches. Also applying the RGAugury pipeline, 566 NLRs, 1517 RLKs and 260 RLPs were predicted in the cultivar ZS11 assembly [50]. 

Fewer NLRs, RLPs and RLKs were predicted by Tirnaz et al. [50] (286 NLRs, 989 RLKs and 77 RLPs) and by Dolatabadian et al. [51] (293 NLRs, 1010 RLKs and 78 RLPs) in the Darmor-*bzh* v8.1 assembly compared to the Darmor-*bzh* v4.1 assembly (621 NLRs, 1497 RLKs and 273 RLPs) [50]. Also, far fewer NLRs, RLPs and RLKs were predicted in Tapidor v6.3 (208, 680 and 223) compared to Darmor-*bzh* v4.1 [50]. This disparity is likely caused by differences in sequencing, assembly and repeat masking approaches [33,54]. Tapidor v6.3 was assembled using Illumina and mate pair reads and scaffolded by comparison to the Darmor-*bzh* v8.1 assembly and its genetic map. In comparison, the Darmor-*bzh* v4.1 assembly was constructed mainly from 454 (>700 bp) and Sanger reads [32]. Resistance genes that are fused with transposable elements could be masked, which may partially explain the difference in the number of NLRs identified [54].

In comparison with predictions based on single genome assemblies, more RGAs with diverse patterns are predicted in the *B. napus* pangenome which was constructed from 24 oilseeds, three fodders, two swedes, two vegetables and 19 synthetic accessions [51]. A total of 503 NLRs, 1098 RLKs and 148 RLPs were identified. Of all these genes, 996 were core and 753 were variable. In addition, the nonsynthetic lines had fewer RGAs (91.87 RGAs per line) than synthetic lines (156.94 RGAs per line). 

### 3.4. B. juncea

A total of 289 NLRs were identified based on the presence of a NB-ARC domain (PF00931) with InterProScan v5.20–59.0 using Pfam 30.0 and coil in the *B. juncea* v1.5 assembly [53]. A total of 1225 RLKs and 228 RLPs were predicted using the RGAugury pipeline [52]. Tirnaz et al. [50] identified 315 NLRs, 1085 RLKs and 191 RLPs using the RGAugury pipeline in the *B. juncea* v1.5 assembly. 

## 4. Reference-Based Candidate RGA Identification

The availability of *Brassica* reference genome assemblies has greatly facilitated QTL mapping and candidate disease resistance gene identification [55]. For example, in a QTL study for sclerotinia stem rot resistance in *B. napus*, the *Brassica* 60K Illumina Infinium array was used to genotype a double haploid (DH) F1 population of the cross between the resistant line J964 and susceptible line J902 [56]. The single nucleotide polymorphism (SNP) markers were anchored onto the *B. napus* Darmor-*bzh* v4.1 assembly and 17 QTL were present on six chromosomes (A02, A09, C02, C03, C04 and C06). The expression of the TNL, *BnaC03g05380D*, which was present in the SRC3a QTL on chromosome C3 was upregulated in resistant plants compared with susceptible plants. 

In a QTL study for clubroot resistance in *B. rapa*, the F2 population of a cross between the susceptible Chinese cabbage and resistant turnip lines was genotyped using double digest restriction site-associated DNA sequencing (ddRAD-seq) and resulting SNP markers were mapped onto the *B. rapa Chiifu-401-42* v.1 assembly [57]. In this study, three NLRs were identified in the new locus, *CRs*, on chromosome A08. In another QTL study for *V. longisporum* resistance in *B. napus*, the *Brassica* 60K Illumina Infinium array was used to genotype an F1 population of the cross Express617 × R53 and a nested association mapping (NAM) panel with five subpopulations [58]. The obtained SNP and single nucleotide absence polymorphism (SNaP) markers were anchored onto the *B. napus* Darmor-*bzh* v4.1 assembly and QTL mapping and genome-wide association studies (GWAS) analysis were performed. QTL for *V. longisporum* resistance were identified on A03, A05, C05, C08 and C09 and seven NLRs within the QTL on C09 were identified. 

Bayer et al. [5] located genetic markers for QTL which were linked with sclerotinia stem rot, black rot, and clubroot in the *B. oleracea* pangenome and the QTL harboured 28 RLKs. Similarly, genetic markers for the QTL for several qualitative blackleg resistance genes including *Rlm1*, *Rlm3*, *Rlm4*, *Rlm7*, *Rlm9*, *LepR1* and *LepR2* were physically located in the *B. napus* pangenome [51]. A total of 60 RGAs were identified in the combined QTL for *Rlm1*, *Rlm3*, *Rlm4*, *Rlm7* and *Rlm9* on chromosome A07, 14 RGAs were located within the *LepR1* QTL on chromosome A02, and 32 RGAs were located within the *LepR2* QTL on chromosome A10.

Sequence comparison of RGAs located within QTL which are associated with disease resistance contributes to candidate gene identification. The TNL allele in the *Crr1a* locus in the resistant line G004 conferred resistance to clubroot isolate Ano-01in *B. rapa* [59]. In comparison, the susceptible allele in the A9709 line encoded a truncated NL protein which lacked more than half of the TIR domain and included 50 substitutions, two deletions, and two insertions. As another example, five NLRs were present in the QTL for TuRB07 which conferred resistance to an isolate of Turnip mosaic virus strain C4 on chromosome A06 in the *B. rapa Chiifu-401-42* v1 assembly [60]. Three NLRs, *Bra018810*, *Bra018834* and *Bra018835*, contained deletions and *Bra018862* was truncated in the resistant line VC1 and Chiifu reference, thus they were not functional. *Bra018863*, a strong candidate for TuRB07, encoded a full-length CNL and there were polymorphisms in the CC, NBS and C-terminal domains in comparison with the allele in the susceptible cultivar SR5.

RGA comparison also contributed to the identification of qualitative blackleg resistance genes. The blackleg resistance gene *Rlm2* was found to be an allelic variant of the *LepR3* RLP locus in *B. napus* [61]. The two *LepR3* lines, Hyola 60 and Surpass 400, contained an identical allele encoding a single-exon coding sequence (CDS) of 2556 bp. The three *Rlm2* lines, Glacier DH24287, Tapidor and Bristol carried identical alleles encoding a single-exon CDS of 2778 bp. Seven *lepR3*/*rlm2 B. napus* lines, Westar N-o-1, Polo, Columbus, Quantum, Ning You, *B. napus* var. *napobrassica* landrace Hvammsrofa and Topas DH16516 shared an allele encoding a single-exon CDS of 2853 bp. The *rlm2*/*lepR3* alleles in the *B. napus* line Marnoo and the *B. juncea* line AC Vulcan were two-exon CDS of 2219 bp and 2218 bp respectively. Two distinct susceptible alleles encoding single-exon CDS of 2916 bp and 2772 bp were identified in *B. rapa* subsp. *sylvestris* 006-1-1 and the *B. rapa* variety Torch respectively.

## 5. RGASeq for More Efficient Candidate Identification 

The conventional approaches to identify causative RGAs are limited in their ability to precisely pinpoint the causative gene. QTL mapping often only enables the identification of a large genomic region which can contain multiple RGAs and is restricted to the genetic recombination present in a biparental mapping population. GWAS overcomes these limitations, but its reliance on a reference genome, particularly if only a single reference is used, means that significant RGA variation from the reference won’t be detected [62]. These constraints result in extensive fine mapping and gene validation in order to confidently determine the causative RGA [63,64]. Resistance gene enrichment sequencing or RenSeq, is a targeted enrichment sequencing workflow, which enables the capture of plant NLR sequences via the hybridisation of RNA baits [65]. Targeted enrichment of RLPs and RLKs is through RLP/KSeq, a recent adaptation of RenSeq [66]. RenSeq and RLP/KSeq (hereafter collectively referred to as RGASeq) significantly reduce genome size and complexity by only enriching RGA-like sequences and excluding the remaining genome [67]. RGASeq can efficiently provide high sequence coverage of the target regions only, without the need for high-coverage whole genome sequencing that would be required to produce a high-quality de novo genome assembly [68]. RGASeq relies on custom biotinylated RNA oligonucleotides, called baits or probes, which are complementary to RGA-like sequences. The target regions that hybridise to the baits are then ‘fished’ out and amplified. RenSeq performed on wheat NLRs produced an enriched library which contained a 1000-fold increase in NLRs compared to other genes [69]. Typically, tens of thousands of baits that show high sequence similarity to RGAs are used in any one reaction to effectively capture the full RGA repertoire. The effectiveness of RGASeq is largely influenced by the design of the baits, as they determine what part of the genome is targeted and enriched. Although only one gene model, ideally from the target species, is required to design a bait library, gene models from a wider range of sources, beyond that of the target species, are often incorporated into the design, for example Steuernagel et al. [69] included predicted NLRs from seven wheat relatives into their bait library design. The inclusion of gene models from closely related species expands the bait library to capture more RGA variation. As shown, many RGAs have been predicted in *Brassica* species, including in the pangenomes of *B. napus* and *B. oleracea* [5,45,51], and in over 30 genomes of various Brassicaceae species [50]. The NLR content of the well-studied *Brassica* relative, *A. thaliana*, has also been extensively catalogued [70]. These resources will help to create a diverse bait library designed from a wide range of closely related species that can be utilised for RGASeq in *Brassicas*.

Crop wild relatives (CWRs) are valuable sources of genetic diversity, as extensive breeding has rendered domesticated species genetically depauperate, meaning cultivated crops contain fewer RGAs and are more vulnerable to disease [71]. Therefore, it is important to explore the RGA diversity in CRWs and landraces to expand breeding resources [72]. The identification of novel RGAs in wild relatives, however, is difficult since most wild species lack reference genomes. Even when a reference genome is available, traditional approaches like GWAS are not able to detect RGAs that significantly differ to the reference, a problem compounded by the fact that *Brassica* RGAs were found to be highly polymorphic and show significant structural variation [5,51]. In response to this limitation, Arora et al. [62] devised an approach that combines association genetics and RenSeq (AgRenSeq), which analyses *k*-mers that differ between resistant and susceptible lines. *K*-mers are subsequences of DNA that are length *k* and can be used for alignment-free sequence analysis enabling association mapping in the absence of a reference genome [73]. Notably, the AgRenSeq approach led to the identification of four wheat stem rust NLR resistance genes, *Sr33*, *Sr45*, *Sr46* and *SrTA1662*, two of which are novel. First, a diverse panel of *Aegilops tauschii*, a wild progenitor of diploid bread wheat, was screened against six races of the wheat stem rust pathogen. The NLR complement of the accessions included in the panel was enriched using a bait library designed for *Ae. tauschii* and was filtered for NLR motifs. After association-based *k-mer* analysis, *k-mers* highly associated with resistance were directly overlaid onto NLR assemblies, revealing full-length stem rust resistance genes. A map-based and mutagenesis approach to identify *Sr46* was also conducted, producing a candidate which was identical to the one found through AgRenSeq, thereby confirming the accuracy and effectiveness of the newly developed AgRenSeq method. 

A similar *k-mer* based association approach could be taken to pinpoint RLPs and RLKs, hence all RGA families could be captured through a combination of association genetics and targeted resistance gene enrichment, which we refer to as AgRGASeq. AgRGASeq can be harnessed to precisely identify causative RGAs in a diverse panel of *Brassica* germplasm in the absence of a reference genome. By utilising *k*-mers, this approach has the potential to capture the full diversity of RGAs, including those that greatly diverge from a reference and would otherwise go undetected. Its application in canola-blackleg resistance could be advantageous, as there are numerous closely related genera containing wild species which show resistance to blackleg, the majority of which have no or limited reference genomes [74,75,76,77,78]. Therefore, AgRGASeq could be used to unlock CWRs untapped RGA resources. 

Steuernagel et al. [69] deployed a different approach to identify and clone causative NLRs in the large and complex hexaploid bread wheat genome, exploiting loss-of-resistance mutations caused by chemical mutagenesis and RenSeq (MutRenSeq). To test MutRenSeq, a previously cloned wheat stem rust NLR, *Sr33* [79], was identified through the enrichment and comparison of NLRs captured from six susceptible mutants with ethyl methanesulfonate (EMS) derived mutations for *Sr33* and a resistant *Sr33* wild-type. Causative mutations were found in the resulting NLR contigs and were identical to the previously discovered mutations. Steuernagel et al. then cloned two uncharacterised wheat stem rust NLRs, *Sr22* and *Sr45*, using a similar method. MutRenSeq offers a fast workflow to pinpoint functional RGAs without fine mapping or positional cloning efforts, given you have the appropriate resistant wild type harbouring only one qualitative resistance gene and loss of disease resistance mutants [80]. To date, no studies have conducted mutagenesis on *Brassicas* with the aim of increasing disease resistance, thus MutRenSeq is a promising modification of RenSeq that could open a new avenue for RGA identification within *Brassica* species. 

One of the dominant long read sequencing platforms, Pacific BioSciences (PacBio) single molecule real time sequencing (SMRT) was used in conjunction with RenSeq (SMRT RenSeq) to identify and clone a late blight broad-spectrum NLR, *Rpi-amr3i*, from *Solanum americanum*, a wild diploid potato relative [81]. Using a mapping population, a bulked segregant analysis approach was coupled with RenSeq to produce an enriched NLR complement of one resistant and one susceptible accession followed by PacBio SMRT sequencing. The consensus long reads were then filtered for NLR-like sequences and six candidates were identified. *Rpi-amr3i* was confirmed as the causative NLR through transgenic expression analysis. Here, SMRT RenSeq was able to obtain high coverage (5–70 X) long reads of potato NLRs, which captured the flanking promoter and terminator sequences, allowing for the assembly of the complete gene. The long reads offered by SMRT RenSeq may be key in overcoming the limitations posed by the large structural variation found in *Brassica* RGAs. 

## 6. Building Pan-RGAomes to Capture Wider RGA Diversity

Due to the recognition that there is large genomic variation within a species, plant pangenomes are becoming widely adopted in plant genomics [38]. In a similar vein, the genome-wide annotation of RGAs from a single reference does not capture the presence/absence and SNP variation in the RGA repertoire within a species. Here, we reason that the pangenome concept can be applied to RGAs, where the RGA repertoire of many individuals of a species, together make up the ‘pan-RGAome’. The pan-RGAome would capture the near-complete species-wide diversity of RGAs and include those that diverge from a single reference. Several methods would enable the construction of a pan-RGAome, including a whole genome resequencing (WGRS) approach, whereby RGAs would be predicted from a pangenome or through the use of RenSeq, which would involve targeting and enriching the RGA complement of many accessions of a species. 

The near-complete species-wide NLR inventory, or pan-NLRome, in the model species *A. thaliana* was recently constructed using SMRT RenSeq and is the first example of defining a species-wide repertoire of plant immunity genes [70]. The NLR complement of 64 diverse *A. thaliana* accessions was enriched using a custom bait library, sequenced and reconstructed, producing 13,167 NLR gene annotations. The pan-NLRome was found to be almost completely defined with the NLRs from any 40 accessions out of the 64 included in the analysis, indicating that even though NLR loci show high variability, there is a finite number of divergent loci. In addition, the creation of the pan-NLRome enabled the identification of new NLR architectures, including some which contained integrated domains. The pan-NLRome for *A. thaliana* serves as a blueprint for future studies that wish to categorize species-wide RGA diversity. Importantly, the development of RLP/KSeq has allowed the capture of RGAs beyond the NLR family and through a combination of RenSeq and RLP/KSeq, building a pan-RGAome using targeted enrichment technology is now possible. 

Although creating a pan-RGAome through RGA prediction in a pangenome is attainable, for example in *B. napus* and *B. oleracea* [5,51], it is limited to the accessions that are incorporated into the pangenome. In this regard, it is well-suited to species that already have reference genomes available for many accessions, usually well-studied major crops. If new lines of interest need to be added to the pan-RGAome, the reference genome of that line would also need to be incorporated into the pangenome before RGA prediction to ensure that any variation would map back to a reference. A RGASeq approach that utilises *k*-mer based analysis, on the other hand, like the one taken by Arora et al. [62], would enable the pan-RGAome in species lacking a reference genome to be defined, especially if used in conjunction with long-read sequencing. Characterising the pan-RGAome of wild *Brassica* relatives that demonstrate strong resistance towards disease would broaden the RGA pool available for improved cultivar breeding.

## 7. Conclusions

*Brassica* crops are threatened by diseases, such as blackleg, sclerotinia stem rot and clubroot. Understanding the genetic basis of disease resistance is crucial for managing these diseases. Construction of multiple *Brassica* genome and pangenome assemblies have greatly facilitated reference-based QTL mapping for disease resistance phenotypes. RGAs are found to be associated with the resistance mechanisms. Genome-wide and pangenome-wide prediction of RGAs and comparison of sequence variations of the RGAs located in previously identified QTL have contributed to efficient identification of RGA candidates. However, reference-based QTL mapping and cloning could be limited when exploring novel resistance in landraces and wild relatives which harbour greater genetic diversity.

Describing the full species-wide diversity of RGAs is an important initial step in identifying causative RGAs and critical in understanding the evolution of disease resistance genes. Building a pan-RGAome provides a comprehensive overview of RGA variation and architecture, which is highly relevant to *Brassica* species as there is significant RGA variation among individuals. It will also prove useful in narrowing down RGAs in previously described QTL by providing the near complete catalogue of potential candidate genes. Finally, defining the pan-RGAome will be essential in understanding the complex evolution of RGAs, which will provide insight into the mechanisms of disease resistance. The pan-RGAome is a promising and powerful resource which will help to improve cultivars through disease resistance breeding.

## Figures and Tables

**Table 1:** Summary of the Genome-wide Prediction of nucleotide-binding-site leucine-rich repeat (NLR), receptor-like kinase (RLKs) and receptor-like proteins (RLPs) in *Brassica* Genome Assemblies.

Species.	Assemblies	NLR	RLK	RLP	Prediction Methods	References for RGA Prediction
*B. rapa*	BAC clones of *B. rapa* ssp. *pekinensis cv. Chiifu*	92	NA	NA	FGENESH program, BLAST and EST sequences	[42]
	*Chiifu-401-42* v1	206	NA	NA	HMMER	[43]
	*Chiifu-401-42* v1	204	NA	NA	BLAST and HMMsearch	[44]
	*Chiifu-401-42* v1	249	NA	NA	MEME/MAST and InterProScan	[45]
	*Chiifu-401-42* v1	202	NA	NA	HMMER for prediction and verified on the Pfam website.	[46]
	*Chiifu-401-42* v1	NA	303	NA	Pfam database	[47]
*B. oleracea*	TO1000 v1	157	NA	NA	HMMER	[43]
	TO1000 v1	443	NA	NA	MEME/MAST and InterProScan	[45]
	TO1000 v1 and *capitata* homozygous line 02–12	146	NA	NA	HMMER for prediction and verified on the Pfam website.	[46]
	*capitata* homozygous line 02–12	138	NA	NA	HMMER	[48]
	TO1000 v2.1	439	822	159	The RGAugury pipeline	[5]
	Pangenome	556	901	213	The RGAugury pipeline	[5]
*B. napus*	Darmor-*bzh* v4.1	425	NA	NA	NA	[32]
	Darmor-*bzh* v4.1	641	NA	NA	MEME/MAST and InterProScan	[45]
	Darmor-*bzh* v4.1	464	NA	NA	HMMER for prediction and verified on the Pfam website.	[46]
	Darmor-*bzh* v4.1	NA	NA	184	MEME/MAST for prediction and verified with Pfam, TMHMM, Phobius and SMART searches	[49]
	Darmor-*bzh* v4.1	621	1497	273	The RGAugury pipeline	[50]
	Darmor-*bzh* v8.1	286	989	77		
	Tapidor v6.3	208	680	223		
	ZS11	566	1517	260		
	Pangenome based on Darmor-*bzh* v8.1	503	1098	148	The RGAugury pipeline	[51]
*B. juncea*	v1.5	289	1225	228	InterProScan and the RGAugury pipeline	[52,53]
	v1.5 Yang_et_al_2016	315	1085	191	The RGAugury pipeline	[50]
